# Somatotype of Competitive Youth Soccer Players From Brazil

**DOI:** 10.2478/hukin-2014-0079

**Published:** 2014-10-10

**Authors:** Yara Lucy Fidelix, Juliane Berria, Elisa Pinheiro Ferrari, Jaelson Gonçalves Ortiz, Tiago Cetolin, Edio Luiz Petroski

**Affiliations:** 1 University of Pernambuco. Associate Program Graduate in Physical Education UPE / UFPB. Recife, Pernambuco, Brazil.; 2 Universidade Federal de Santa Catarina. Postgraduate Program in Physical Education. Florianópolis, Santa Catarina, Brazil.; 3 State University of Santa Catarina (UDESC), Center for Health Sciences and Sports, Graduate Program in Human Movement Science. Florianópolis, Santa Catarina, Brazil.

**Keywords:** athletes, anthropometry, male, soccer, youth

## Abstract

The aim of this study was to identify the morphological configuration of youth athletes from professional soccer clubs and to verify their differences according to the tactical position on the field. Overall, 67 male players aged 15 to 17 years were evaluated. The examined anthropometric measurements included body mass, body height, skinfolds (triceps, subscapular, supraspinal and medial calf), girths (flexed and tensed arm and calf) and breadths (humerus and femur). For statistical purposes, analysis of variance and post hoc Bonferroni and Kruskal-Wallis tests were used. We concluded that goalkeepers were heavier and taller than center backs (p = 0.015 and p = 0.001), midfielders (p = 0.005 and p <0.001) and center forward players (p = 0.024 and p <0.001). The average somatotype for defense, forward and goalkeeper positions was a balanced mesomorph. Midfield players showed ectomorphic-mesomorph characteristics. It was concluded that goalkeepers were characterized as being taller and heavier and that somatotype features of athletes were similar between positions, except for midfield players.

## Introduction

High-performance sport is constantly searching for ways to improve results, win competitions and break records. The search for as well as the identification and orientation of talents are considered a concern in high-performance sport (Borin and Gonçalves, 2008). Soccer is the most popular sport in Brazil and has thousands of practitioners worldwide.

The physiological, anthropometric and neuromuscular characteristics of sport teams vary according to each discipline (Gobbo et al., 2002). Coaches generally report that technical and tactical aspects are extremely important for performance (Souza and Zucas, 2003); however, great importance has been ascribed to the morphological characteristics of players because they can be considered the basis of technical and tactical development (Chamari et al., 2004). Some factors such as heredity, nutritional aspects and physical training can significantly contribute to the success of an athlete, especially in high-performance sport (Gobbo et al., 2002).

Research has shown that athletes differ from the general population regarding body composition, and these differences are also observed between starting players and substitutes, between playing positions and a competition level (regional, national or international) (Nikolaidis and Karydis, 2011; Hazir, 2010; Moghadam et al., 2012). The study by Bandyopadhyay (2007) indicated that while the general population presents a fat content of 16.7%, male athletes have a mean value of 10%. In elite soccer players, differences were identified among goalkeepers, full back, midfield and forward players in terms of mean fat content and body mass values (p <0.001) (Carling and Orhant, 2010). Evaluating 241 players from a Spanish club, Gil et al. (2007) found that forward players were generally thinner and had higher fat free mass, and they performed better in speed, strength, agility and power tests. In contrast, goalkeepers were taller, heavier and had a higher fat content.

High-performance teams continuously seek strategies that may be useful in selecting new talents. A tool that can be used in this type of “diagnosis” is the somatotype. Although body shape and size are not the only variables necessary to determine the success of an athlete, they may represent important prerequisites for certain sports (Gualdi-Russo and Zaccagni, 2001). Somatotype identification allows the development of specific training programs for each physical characteristic, which differs between sports, positions and game requirements. It also allows the verification of differences between athletes who practice the same sport, differentiating them based on their adiposity level, robustness and musculoskeletal linearity (Carter and Heath, 1990).

Studies related to the somatotype of juvenile players are poorly explored, and this can be explained, in part, by the difficulty researchers encounter in accessing professional soccer clubs and also by the difficulty of data collection in terms of training schedules, which are usually held in two day shifts, which are not characteristics of amateur teams.

The best performances are observed for individuals who have the anatomical and morphological characteristics more favorable for specific sport, considering the anthropometric characteristics as part of the set of biological variables related to sports performance (Knechtle et al., 2011). Thus, body morphology is an important factor for both the identification of sports talents and the development of professional athletes (Landers et al., 2000).

In this context, the objective of this study was to identify the somatotype of juvenile athletes from professional soccer clubs and to verify the somatotype differences according to their playing positions.

## Material and Methods

This study on the somatotype analysis of youth athletes from professional soccer clubs was developed from a cross-sectional study entitled “Morphological analysis of professional soccer players”, approved by the Ethics Committee on Human Research of the Federal University of Santa Catarina (UFSC), process number 144/09.

### Participants

The study population consisted of 67 male youth soccer players aged 15 to 17 years from two professional soccer clubs in southern Brazil. The athletes were in the preparatory period for competitions, practicing six days a week, with training sessions divided into three mesocycles (general stage, specific stage and pre-competitive stage) and subdivided into six microcycles, which lasted two weeks each, totaling 12 weeks of training. During microcycles 1 and 2, aerobic and anaerobic capacity as well as strength endurance were developed, while in microcycles 3 and 4, predominant activities included maximal strength and explosive strength in addition to aerobic power maintenance, speed and speed endurance. Microcycles 5 and 6 were aimed at developing speed and speed endurance as well as the maintenance of maximal strength and speed.

### Measures

In a previously prepared room located in the football club’s facilities, anthropometric measurements of body mass, body height, skinfolds (SF) (triceps, subscapular, supraspinal and medial calf), flexed and tensed arm girth and calf girth, humerus breadth (biepicondylar) and femur breadth (biepicondylar) were conducted. These measurements were performed according to procedures proposed by ISAK (Marfell-Jones et al., 2006), with two non-consecutive measurements for each location. In case of a measurement difference exceeding 5% in two non-consecutive measurements, a third measurement was performed, and the mean of the two closest measurements was used.

Body mass was measured using a Filizola ® digital scale with accuracy of 0.1 kg and body height was measured with a Sanny ® stadiometer, with accuracy of 0.1 cm. Skinfolds were measured with a Cescorf ® scientific caliper with accuracy of 0.1 mm, and girths were measured with a Cescorf ® flexible anthropometric tape with accuracy of 0.1 mm.

Body mass and body height measurements were used to calculate the body mass index (BMI). For the calculation of somatotype components, the equations of Carter and Heath were used (Carter and Heath, 1990).

### Procedures

Data collection was conducted in April 2012 on the premises of the soccer clubs in the morning shift, before the start of training. The team of evaluators was composed of 13 physical education students and teachers. Eight evaluators conducted anthropometric measurements (two for skinfolds, two for girths and two for diameters, one for body mass and one for body height). Evaluators who performed the anthropometric measurements were certified by the International Society for the Advancement of Kinanthropometry (ISAK).

Due to a small number of athletes for data analysis, the following playing positions were grouped: backs and defenders formed the defense group, although they had specificities related to their playing position, both could act as defenders; midfielders and forwards formed the midfield group.

### Statistical Analysis

Initially, descriptive statistics were used, with data presented as the mean and standard deviation to characterize the sample, according to the playing position. The normality of the data was analyzed using the Kolmogorov Smirnov test. Normal distribution was identified for body mass, body height, BMI, flexed and tensed arm girth and calf girth, triceps SF, femur breadth. Age, subscapular SF, supraspinal SF, medial calf SF, and humerus breadth showed no normal distribution even after log_10_ transformation. Analysis of variance (ANOVA) and the *post hoc* Bonferroni test were used to compare anthropometric variables according to the playing position. For age, the Kruskal-Wallis test was used. The significance level was set at 5%. Data were entered into Excel ® and analyzed with the Statistical Package for Social Sciences (SPSS Inc., Chicago, IL) version 15.0. To identify the somatotype differences between positions, Somatotype Attitudinal Distance (SAD) was used, which is characterized as the spatial distance between two somatopoints and identifies significant differences from SAD> 2.0 (Hebbelinck et al., 1975).

## Results

The general characteristics of youth soccer players according to the playing position are described in Table 1. Differences were observed for body height (p <0.001) and body mass (p = 0.008); goalkeepers were heavier and taller than defense (p = 0.015 and p = 0.001), midfield (p = 0.005 and p <0.001) and center forward players (p = 0.024 and p <0.001). There were differences in the anthropometric measurements of arm circumference (p = 0.010), triceps SF (p = 0.001) and medial calf SF (p = 0.046). The arm circumference of goalkeepers was greater than that of defense players (p = 0.028) and midfield players (p = 0.007). The triceps skinfold of goalkeepers was higher than that of midfield (p = 0.001) and center forward players (0.014), and that of defense players was higher compared to midfield players.

Table 2 shows the mean values for each somatotype component and classification. The mean somatotype for defense and forward positions as well as goalkeepers was a balanced mesomorph. Midfield players showed ectomorphic-mesomorphic characteristics.

For best viewing the somatotyping results, the values were plotted in the somatochart, in which diamonds represent the athletes and the circle refers to the location of the mean somatotype in the population investigated.

**Figure 1:**
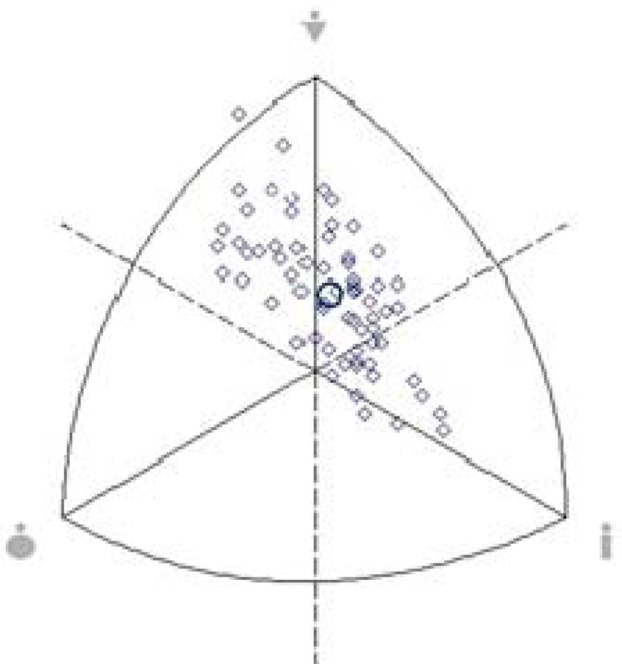
Somatochart with the mean somatotype values of juvenile soccer players. ⋄: athletes; ○: mean of the population investigated. Endomorphy is located on the left, mesomorphy at the top and ectomorphy on the right of the somatochart.

Figure 1 shows that the mean somatotype of juvenile athletes was a balanced mesomorph (2.6 - 4.3 - 2.9).

**Figure 2:**
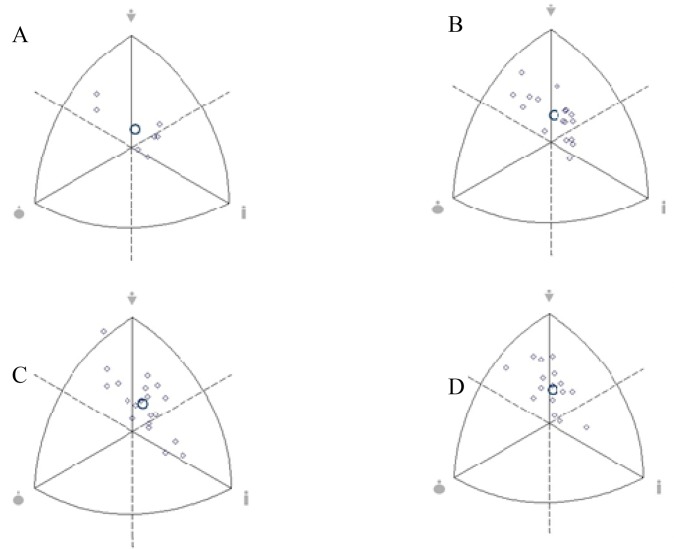
Somatochart of goalkeepers (A) and defense (B), midfield (C) and forward players (D). ⋄: athletes; ○: mean of the population investigated.

Figure 2 shows the somatotype of athletes for the following playing positions: goalkeeper, center back, midfield and forward. Only midfield players had a different classification and were characterized as ectomorphic mesomorphs, while all other positions were classified as a balanced mesomorph.

## Discussion

Goalkeepers had higher body mass and were taller than midfield and forward players; this result is similar to those of other studies (Gil et al., 2010; Wong et al., 2009) in amateur Spanish goalkeepers, who were taller, heavier and had higher fat content in comparison to other positions (Gil et al., 2010).

The average somatotype of athletes evaluated in this study was 2.6-4.3-2.9, showing a high mesomorphy value. This was observed in other studies on Gaelic professional players (Watson, 1995), elite Nigerian athletes (Mathur et al., 1985), professional and semiprofessional Brazilian athletes (Castanhede et al., 2003; Ribeiro et al., 2007) as well as Spanish athletes (Casajús and Aragonés, 1991).

Somatotype components change throughout adolescence (Nikolaidis and Karydis, 2011), with a slight increase in mesomorphy among boys around the age of 13 years (Malina et al., 2004). A previous study investigating the somatotype of 203 players aged between 14 and 19 years of U15 (2.5-4.2-3.4), U16 (2.3-4.3-3.1), U17 (2.6-4.4-2.6), U18 (2.5-4.4-2.6) and U19 (2.4-4.3-2.4) categories found a decrease in the ectomorphy component with advancing age (Gil et al., 2010).

Another study on adolescent soccer players showed a positive association between age and mesomorphy, i.e., older adolescents showed greater muscle development (Bandyopadhyay, 2007).

Youth soccer players in this study were classified as balanced mesomorphs, featuring a predominance of a muscle skeletal component and a balance of fat and linearity components. Soccer athletes of teams in Rio de Janeiro, participants of the 2001/2002 first division of the Brazilian league also showed the ectomorphicmesomorph characteristic (2.7-4.8-2.3) (Castanhede et al., 2007). The same classification was observed in North American (Galavíz and Fierro, 2007) and South American athletes (Rienzi et al., 2000), although the ages differed between these studies. Based on these results, it can be inferred that there is homogeneity among athletes, despite different playing positions. This can be explained by the fact that athletes undergo a selection process to join clubs, and those who do not have specific physical characteristics for this sport are automatically excluded.

Considering the tactical positions played in the match, the morphological configuration of center back and forward players, as well as goalkeepers, was classified as a balanced mesomorph, while midfield players were classified as ectomorphic mesomorphs, whose skeletal muscle component was dominant followed by the linearity component. The findings of this study differ from those in the literature, which shows that goalkeepers have different somatotype characteristics compared to other positions (Rienzi et al., 2000), presenting higher mean endomorphy values (Casajús and Aragonés, 1991).

In the present study, the only players who showed characteristics different from other positions somatotype were midfield players. The distance traveled by midfield players is significantly higher than that of backs and forwards (Reilly et al., 2000), suggesting that this playing position requires a higher level of aerobic capacity (Reilly, 1997), which is strongly influenced by fat-free mass (Goran et al., 2000). Furthermore, the position of each specific training should be considered (Gil et al., 2007).

The balanced mesomorph somatotype has been observed for most positions in national and international studies. This could be observed in South American professional athletes, where the somatotype characteristics were the same for all positions except for goalkeepers (Rienzi et al., 2000). Ribeiro et al. (2007) investigated only full back / wing back players in the U20 category of Minas Gerais and found that both professional and semiprofessional athletes were classified as balanced mesomorphs, with somatotype values of 2.4-4.4-3.0 and 2.7-4.5-3.2, respectively. A similar result was found for soccer players in the United States, who, regardless of the playing position, were classified as balanced mesomorphs (Galavíz and Fierro, 2007).

The difference in somatotype according to the playing position analyzed using the SAD, which identifies the dispersal distance between two somatopoints, establishing a significant distance when SAD > 2, was found between goalkeepers and midfield players (SAD = 2.4). Analysis of SAD is not able to discriminate where the difference occurs; therefore, it is only used to indicate that the somatotypes compared are not similar, showing that there are differences between the midpoints plotted in the somatochart for these groups.

The possible selection bias is among the study’s limitations because our sample was obtained by convenience, indicating that the somatotype profile of athletes in this study can be specific to the clubs where they train because these characteristics may vary according to the club size, geographical location, among others. Thus, it is noteworthy that the profile of athletes from professional clubs may be different from that of amateur athletes, as they have distinct training and monitoring conditions (specialized training, nutritionists, etc.). However, the lack of data in the literature regarding the somatotype of juvenile athletes limited the comparison of results. In this context, the present study contributes to the existing literature, providing information about the somatotype characteristics of juvenile athletes of a professional soccer club. The analysis was performed according to the playing position.

According to the results of this study, it was concluded that goalkeepers showed higher values for the variables of body mass and height compared to other playing positions. Furthermore, the somatotype characteristics of athletes are similar between positions, except for midfield players. This information is useful for coaches and professionals involved in sports, as it can be used in the process of talent selection and the development of training programs because they serve as a reference for athletes of the same sex, age and competitive level.

## Figures and Tables

**Table 1:** General characteristics of youth soccer players according to their position

	***Goalkeeper* (n=07)**		***Defense*** (n=22)		***Midfield*** (n=20)		***Forward*** (n=18)	

	Mean	SD	Mean	SD	Mean	SD	Mean	SD
Age (years)^†^	16.29	0.76	16.09	*0.75*	*16.40*	*0.68*	*16.17*	*0.79*
Body Mass (kg)	80.5^*^	4.26	69.9	*7.89*	*68.6*	*6.97*	*70.2*	*9.20*
Body Height (cm)	1.88^*^	2.63	177.6	*6.52*	*175.9*	*5.78*	*175.8*	*6.89*
BMI (kg/m^2^)	22.80	1.61	22.12	*1.76*	*22.15*	*1.87*	*22.63*	*1.74*
Girths (cm)								
Relaxed arm girth	33.4^*^	1.56	30.9	*1.61*	*30.5*	*1.88*	*31.4*	*2.52*
Calf girth (max.)	38.0	2.16	37.0	*2.21*	*36.4*	*2.22*	*36.7*	*1.96*
Skinfolds (mm)								
Triceps	11.0^*^	3.48	9.3^*^	*2.23*	*7.3*	*1.67*	*8.0*	*1.96*
Subscapular^†^	10.4	3.69	8.7	*1.64*	*8.4*	*1.71*	*9.0*	*1.62*
Supraspinal^†^	8.9	2.55	8.9	*2.87*	*7.5*	*2.01*	*7.9*	*2.58*
Medial Calf^†^	9.5	2.58	7.3	*2.04*	*6.4*	*1.35*	*7.0*	*2.74*
Breadth (mm)								
Humerus^†^	7.1	0.51	6.7	*0.65*	*6.5*	*0.64*	*6.8*	*0.50*
Femur	10.2	0.49	9.8	*0.66*	*9.5*	*0.73*	*9.7*	*0.65*

Defense: center back + full back players; Midfield: Midfield + center forward players SD: standard deviation, kg: kilograms, cm: centimeters, m: meters;

† Kruskal-Wallis test;

* highest average value; Body Mass: goalkeepers ≠ defense players (p = 0.015); goalkeepers ≠ midfield players (p = 0.005); goalkeepers ≠ center forward players (p = 0.024); Height: goalkeepers ≠ defense players (p = 0.001); goalkeepers ≠ midfield players (p = 0.001); goalkeepers ≠ center forward players (p = 0.001), C: circumference (in centimeters); SF: skinfold (mm), D: diameter (in centimeters); C. Arm: goalkeepers ≠ defense players (p = 0.028); goalkeepers ≠ midfield players (p = 0.007); Triceps SF: goalkeepers ≠ midfield players (p = 0.001); goalkeepers ≠ center forward players (p = 0.014); defense players ≠ midfield players (p = 0.019).

**Table 2:** Mean somatotype and classification of youth athletes according to their position

	Mean somatotype			Classification
	Endo	Meso	Ecto	
Goalkeeper	3.1	4.1	2.9	balanced mesomorph
Defender	2.7	4.2	3.0	balanced mesomorph
Midfielder	2.3	4.1	2.9	ectomorphic mesomorph
Forward	2.5	4.7	2.6	balanced mesomorph
Mean	2.6	4.3	2.9	balanced mesomorph
